# pRAD50: a novel and clinically applicable pharmacodynamic biomarker of both ATM and ATR inhibition identified using mass spectrometry and immunohistochemistry

**DOI:** 10.1038/s41416-018-0286-4

**Published:** 2018-11-02

**Authors:** Gemma N. Jones, Claire Rooney, Nicola Griffin, Martine Roudier, Lucy A. Young, Antonio Garcia-Trinidad, Gareth D. Hughes, Jeffrey R. Whiteaker, Zena Wilson, Rajesh Odedra, Lei Zhao, Richard G. Ivey, William J. Howat, Elizabeth A. Harrington, J. Carl Barrett, Antonio Ramos-Montoya, Alan Lau, Amanda G. Paulovich, Elaine B. Cadogan, Andrew J. Pierce

**Affiliations:** 1 Translational Sciences, Oncology, IMED Biotech Unit, AstraZeneca, Cambridge, UK; 2 Oncology Bioscience, IMED Biotech Unit, AstraZeneca, Cambridge, UK; 3 Clinical Research Division, Fred Hutchinson Cancer Research Centre, Seattle, WA USA

## Abstract

**Background:**

AZD0156 and AZD6738 are potent and selective inhibitors of ataxia-telangiectasia-kinase (ATM) and ataxia-telangiectasia-mutated and Rad3-related (ATR), respectively, important sensors/signallers of DNA damage.

**Methods:**

We used multiplexed targeted-mass-spectrometry to select pRAD50(Ser635) as a pharmacodynamic biomarker for AZD0156-mediated ATM inhibition from a panel of 45 peptides, then developed and tested a clinically applicable immunohistochemistry assay for pRAD50(Ser635) detection in FFPE tissue.

**Results:**

We found moderate pRAD50 baseline levels across cancer indications. pRAD50 was detectable in 100% gastric cancers (*n* = 23), 99% colorectal cancers (*n* = 102), 95% triple-negative-breast cancers (TNBC) (*n* = 40) and 87.5% glioblastoma-multiformes (*n* = 16). We demonstrated AZD0156 target inhibition in TNBC patient-derived xenograft models; where AZD0156 monotherapy or post olaparib treatment, resulted in a 34–72% reduction in pRAD50. Similar inhibition of pRAD50 (68%) was observed following ATM inhibitor treatment post irinotecan in a colorectal cancer xenograft model. ATR inhibition, using AZD6738, increased pRAD50 in the ATM-proficient models whilst in ATM-deficient models the opposite was observed, suggesting pRAD50 pharmacodynamics post ATR inhibition may be ATM-dependent and could be useful to determine ATM functionality in patients treated with ATR inhibitors.

**Conclusion:**

Together these data support clinical utilisation of pRAD50 as a biomarker of AZD0156 and AZD6738 pharmacology to elucidate clinical pharmacokinetic/pharmacodynamic relationships, thereby informing recommended Phase 2 dose/schedule.

## Introduction

Ataxia-telangiectasia-mutated (ATM) is an attractive therapeutic target as constitutional loss of ATM in ataxia-telangiectasia (A-T) patients causes profound sensitivity to DNA damaging agents that induce double-strand breaks (e.g. ionizing radiation).^1,2^ ATM is the major signalling kinase involved in the initiation of double-strand break (DSB) repair by homologous recombination (HR) and activated by exposed DNA double-stranded ends.^3^ ATM can trigger cell cycle checkpoints (G1/S and intra-S phase) to ensure fidelity of DNA repair and cell survival.^4^

The functions of the MRE11, RAD50 and NBS1 (MRN) complex are critical to DNA double-stranded end repair and ATM activation.^5^ The MRN complex facilitates recruitment of an inactive ATM dimer to exposed double-strand DNA ends, the interaction results in a conformation change of ATM to an open active ATM dimer and autophosphorylation of ATM at Ser1981 (Supplementary Figure 1).^6^ Activated ATM then phosphorylates its downstream substrates, including all three components of the MRN complex, which are known to have distinct roles in signal transduction to the DNA repair machinery, cell cycle checkpoint controls and cellular processes.^3,7^ Diminished levels of MRE11 functionality, for example from patients with hypomorphic mutations, result in patients with an A-T-like disorder (ATLD), presenting with radiation sensitivity and chromosomal instability.^8^ Nijmegen breakage syndrome (NBS) patients that have a hypomorphic mutation in the gene encoding NBS1 and are also deficient in ATM signalling as MRE11 and RAD50 fail to correctly localise in the nucleus.^9,10^ RAD50 deficiency is termed NBS-like disorder due to the similar clinical features to NBS, including radio-sensitivity, chromosomal instability, cell cycle abnormalities and impaired ATM signalling.^11^ RAD50 deficiency in the clinic is rare, with one patient found to have a compound heterozygous RAD50 mutation,^11^ whilst another presented with a clonal hemizygous mutation in RAD50 and extreme sensitivity to irinotecan in combination with a CHK1 inhibitor.^12^

ATM and Ataxia-telangiectasia-mutated and Rad3-related (ATR) are both phosphatidylinositol-3 kinase-related kinases (PIKKs) under investigation as therapeutic targets in oncology. In contrast to ATM, ATR is the apical kinase of the DNA replication stress response and is recruited to and activated by the interaction of ATR interacting protein (ATRIP) with replication protein A (RPA), bound to extended single-stranded DNA at stalled replication forks. This interaction causes stabilisation of the stalled forks, suppression of further replication origin firing and cell cycle arrest (intra-S and G2/M phases).^13^ Hypomorphic mutations in human ATR cause the rare autosomal-recessive disease Seckel syndrome, and complete loss of ATR in mice leads to embryonic lethality, although partial suppression is compatible with life.^14^ However, small molecule inhibition of ATR in cell lines results in enhanced sensitivity to radiation, although to a lesser extent than ATM inhibition.^15,16^

AZD0156 and AZD6738 are potent and selective orally bioavailable ATP competitive inhibitors of ATM and ATR, respectively. AZD0156 is in early clinical trials in combination with the PARP inhibitor, olaparib (Lynparza), as well as other DNA damage-inducing agents [NCT02588105],^17^ while AZD6738 is in clinical trials as a monotherapy and in combination with olaparib (Lynparza), anti-PDL1 durvalumab (Imfinzi), ionising radiation and chemotherapy [NCT01955668, NCT03022409, NCT02264678, NCT02223923, NCT02630199].^16,18^ By utilising these compounds in the presence of either endogenous or exogenous DNA damage, it is expected that DNA damage within the cancer cells will not be resolved, resulting in cell death and tumour regression.^19^

Development of robust pharmacodynamic (PD) biomarkers is needed in clinical trials to establish the relationship between drug exposure and PD modulation, thereby facilitating determination of an appropriate recommended Phase 2 dose (RP2D)/schedule, consistent with good practice in oncology early clinical development.^20^ Here we used immuno-multiple-reaction monitoring mass spectrometry (immuno-MRM-MS) to quantitatively identify a clinically applicable PD biomarker that could inform modulation of ATM signalling by AZD0156. Immuno-MRM-MS is a technique that combines immunoaffinity enrichment of peptides with the high sensitivity and selectivity of MRM-MS detection.^21^ The approach has gained considerable interest in recent years due to several advantages over traditional protein measurement techniques. As the analyte is directly detected using the mass spectrometer, cross-reactivity of the antibodies used for enrichment do not affect assay specificity. Furthermore, a single antibody can be employed to enrich the phosphorylated and unmodified versions of the same peptide, using the mass spectrometer to distinguish between the two. Precise quantification and standardisation of results across laboratories is possible with MRM-MS, as it uses synthetic stable isotope-labelled peptides as internal standards,^22^ and assays are readily multiplexed by combining antibodies together for peptide enrichment.^22^

Using the MRM-MS technology, we show how Ser635 phosphorylated RAD50 (pRAD50) can be utilised as a PD biomarker for the AZD0156 clinical trial. We demonstrate in preclinical models that AZD0156 can abrogate the phosphorylation of RAD50, induced by DNA-damaging agents such as ionising radiation, irinotecan and olaparib. We also demonstrate the potential of pRAD50 to be used as a PD biomarker for the AZD6738 clinical trials. We highlight for the first time that modulation of pRAD50 by AZD6738 may depend on the underlying functionality of the ATM pathway in cells, and could be used to determine ATM functional status following AZD6738 treatment.

## Materials And Methods

### PBMC isolation

Blood was collected from four healthy volunteers at Wythenshawe MEU in cell preparation tubes (CPT; BD) and PBMCs were isolated by centrifugation within 2 h of collection. PBMCs were incubated for 1 h at 37 °C in RPMI culture media (Sigma) ± 30 nM AZD0156, then given 5 Gy irradiation using an X-Rad irradiator (Pxi). Cells were subsequently lysed in 6 M Urea, 25 mM Tris (pH8.0), 1 mM EDTA, 1 mM EGTA lysis buffer with phosphatase inhibitor cocktails 1 and 2 (Sigma) and protease inhibitors (Sigma). Lysate protein concentration was determined by BCA quantification (ThermoFisher). Lysates were reduced, alkylated with iodoacetamide, and digested with trypsin overnight at 37 °C. Formic acid was used to quench the reaction, a mix of stable isotope-labelled peptide standards were added prior to desalting using Oasis HLB 96-well plates and a positive pressure manifold (Waters).

### Mass spectrometry

To ensure adequate sample, the PBMC lysates from the four healthy volunteers were pooled into one sample, which was run in process triplicate (including trypsin digestion). Lysates were analysed using immuno-MRM-MS that was multiplexed to quantify a panel of peptides related to the DNA damage response (DDR) (see Supplementary Table 1 for peptide list). Enrichment was performed as previously described^23^ with the following modifications. A mixture of 45 antibodies were individually coupled to magnetic Protein G beads, and 1 μg per antibody-protein G beads was used for enrichment. Enrichment was performed in PBS + 0.03% CHAPS and peptides were eluted in 5% acetic acid/3% acetonitrile. LC-MRM was performed using an Ultra nanoLC system (Eksigent Technologies) with a nano auto-sampler coupled to a 6500 QTRAP mass spectrometer (SCIEX). Mobile phases were 0.1% formic acid in water (A) and 90% acetonitrile/0.1% formic acid in water (B). Peptides were loaded on a trap column at 5 µL/min and 3% B and the LC gradient was delivered at 300 nL/minute consisting of a linear gradient of mobile phase B developed from 3–40% B in 15 minutes on a 10cmx75µm column (Reprosil C18). The hybrid triple quadrupole/ion trap mass spectrometer (6500 QTRAP) was equipped with a nano electrospray interface operated in the positive ion MRM mode. Scheduled MRM transitions used a retention time window of 100 s and a desired cycle time of 0.5 s, enabling sufficient points across a peak for accurate quantitation. Parameters for collision energy (CE) were taken from a linear regression of previously optimised values in Skyline. Raw peak areas were integrated by Skyline and reviewed manually to confirm transitions from analyte and standard peptides coeluted and had consistent relative areas. Total peak areas were calculated using Peak Area + Background and peptide concentrations were determined relative to the amount of spiked internal standard peptides. The assays accurately quantify the reproducibly released tryptic peptides, although this may not accurately represent the full-length protein, depending on the recovery following protein extraction and trypsin digestion. Data were filtered for limits of quantification (LoQ) determined in previous analytical characterisation experiments.^24^ See Supplementary Table 1 for LoQ values for each peptide. The mean concentration of each analyte in fmol/mg was calculated and plotted following *an asinh* transformation.^25^ Antibodies used in the immuno-MRM-MS analysis are available for research use, as are additional details concerning the analytical methods.^26,27^

### Cell culture and DNA damage induction

Cell lines were purchased from ATCC, unless otherwise specified. Cell lines have identities verified by short tandem repeat (STR) analysis and are regularly checked to ensure no mycoplasma contamination. HeLa and FaDu cells were cultured in high glucose DMEM medium (Gibco LifeTech), and NCI-H23 cells were cultured in RPMI medium supplemented with 2 mM L-glutamine (Sigma). All media were supplemented with 10% fetal bovine serum (ThermoFisher). The triple ATM knock-out (KO) FaDu cell line was generated by AstraZeneca (Discovery Sciences, Sweden) using zinc finger nucleases (ZFNs) to knock-out all three alleles of ATM. SilenciX HeLa cells obtained from Tebu-Bio, were cultured in medium supplemented with 125 µg/ml hygromycin B (Invitrogen) to maintain selection of the shRNA plasmid expressing cells. DNA damage was induced in cells by X-ray with 5 or 6 Gy (130 kV, 5 mA) (Faxitron CellRad Irradiator) or by treatment with 1 µM aphidicolin. NCI-H23 cells were treated with a combination of 1 µM aphidicolin and AZD6738 at 0.3 µM or 1 µM in DMSO, or treated with DMSO (Vehicle) or AZD6738 alone.

### Formalin fixed paraffin embedded (FFPE) cell block preparation

Cells were washed in PBS and fixed for 5 min on the cell plate with 10% buffered formalin, then scraped and left in formalin overnight. Cells were washed in PBS and 70% ethanol, then resuspended in warm Histogel (ThermoFisher) to make a cell pellet. The histogel pellet was processed through graded alcohols, xylene and paraffin wax, before being embedded in paraffin using standard methods.

### Animal studies

Xenograft studies were run in the UK in accordance with UK Home Office legislation, the Animal Scientific Procedures Act 1986 (ASPA) and with AstraZeneca Global Bioethics policy. Cultured A549 and SW620 cells were implanted subcutaneously in nude mice in serum-free media with Matrigel (Envigo UK and Harlan UK, respectively). Cultured ATM KO FaDu cells were implanted without Matrigel in SCID mice (Charles River, UK). A549-implanted mice were dosed with oral HPMC/Tween vehicle, 1 h post vehicle dosing one group was irradiated (2Gy), tumours were collected 1 h post-irradiation. SW620-implanted mice were treated with irinotecan (50 mg/kg intraperitoneal) or AZ31 (100 mg/kg oral) in combination or with a vehicle control, 3 h prior to tumour collection. ATM KO FaDu-implanted mice were treated with AZD6738 (50 mg/kg oral) or the vehicle control. Tumours were collected at 3, 8 and 24 h post-dosing; no 8 h time point was collected for the vehicle control.

Triple-negative-breast cancer HBCx-10 and HBCx-9^28^ patient-derived xenograft studies were carried out at XenTech, France in accordance with French regulatory legislation. Female athymic nude mice (Harlan France) were implanted with HBCx-10 or HBCx-9 tumour derived from a primary ductal adenocarcinoma. Donor mice were sacrificed to provide tumour fragments, which were surgically implanted subcutaneously. HBCx-10 implanted mice were dosed once daily for 3 days with olaparib (50 mg/kg oral qd) or AZD0156 (5 mg/kg oral qd) alone or in combination, and samples were taken at 2 h or 24 h post the final olaparib dose. HBCx-9 implanted mice were treated for 3 days with AZD0156 (5 mg/kg oral qd) or 5 days with AZD6738 (25 mg/kg oral qd) or the vehicle control, the 3- and 5-day schedules being selected to align with the anticipated clinical schedules of administration for these compounds. Samples were collected at 3, 7 and 23 h post AZD0156 dose or 2, 8 and 24 h post AZD6738 or vehicle doses. Upon collection, tumours were divided and half was snap frozen for western blot analysis and half was fixed in formalin and embedded in paraffin for IHC analysis.

### Western blot

Cells were collected by scraping or trypsinisation and lysed in RIPA lysis buffer (ThermoFisher) with added phosphatase and protease inhibitors (Sigma). For xenograft tissue, tissue was homogenised in Tris buffer containing 10% glycerol, sodium-orthovanadate and sodium-fluoride, and supplemented with phosphatase and protease inhibitors (Sigma). A BCA assay was used to quantify the lysate protein concentration. Protein electrophoresis was carried out on a 4–12% SDS-PAGE Bis-Tris MidiGel (LifeTech), transferred to a nitrocellulose membrane (iBLOT), then probed for the following primary antibodies diluted in 4% milk in TBS-Tween(0.05%); pATM(Ser1981)[EP1890Y], ATM[Y170], pATR(Thr1989)(GTX128145 GeneTex), ATR(SantaCruz N-19), pCHK1(Ser345)[133D3], CHK1[2G1D5], pCHK2(Thr68)(CST#2661), H2AX(Millipore 07–627), γH2AX[JBW301], RAD50[13B3/2C6], Vinculin [VIN-11–5], and GAPDH[14C10]. pRAD50(Ser635)(CST#14223) antibody was diluted in 5% BSA in TBS-Tween(0.05%). HRP linked goat anti-mouse and goat anti-rabbit secondary antibodies (GEHealthcare] were then applied, followed by SuperSignal West Dura ECL detection reagents (ThermoFisher). Protein samples from cell lines were developed using either the G box (Syngene) or standard film and bands were quantified by Image J. For analysis of xenograft tissue, membranes were developed on the Chemigenius imaging platform to allow quantification of the bands. Western blot gel band intensities were normalised to vinculin unless otherwise noted with samples randomised across individual gels and/or an internal loading control used to normalise inter-gel variability.

### Immunohistochemistry (IHC)

Four micrometers of sections of FFPE tissues was used for IHC. IHC was performed on the Ventana Discovery Ultra (Roche), which involved EZ prep deparaffinisation, followed by CC1 antigen retrieval at 98 °C (64 min for γH2AX, 32 min for pRAD50). Slides were then protein blocked using Signal Stain® diluent (CST) for γH2AX or diluent with casein (Roche) for pRAD50, followed by endogenous peroxidase blocking. Primary antibodies used were phospho-histone H2A.X (Ser139) (γH2AX) 20E3 rabbit monoclonal antibody (CST) at 2.68 µg/ml in TBS-Tween (0.05%), or Ser635 phosphorylated RAD50 (pRAD50) rabbit polyclonal antibody (CST) at 1 µg/ml in PSS antibody diluent (Roche). The detection steps involved anti-Rabbit HQ, anti-HQ HRP, then DAB staining (Roche; Discovery Chromomap DAB kit). Haematoxylin for γH2AX and Haematoxylin II for pRAD50 with bluing reagent (Roche) was used to counterstain the nuclei. Antibody diluent (Buffer control) or matched concentration rabbit DA1E IgG isotype control (CST) was used in place of the primary antibody step for staining negative controls.

### Dephosphorylation

FFPE tissue sections were deparaffinised and antigen retrieved on the Ventana Discovery Ultra as described above. Slides were then incubated for 1 h at 37 °C with control CutSmart buffer or alkaline phosphatase (CIP) enzyme (NEB) to dephosphorylate the tissue, before continuing with the standard IHC staining protocol.

### IHC image analysis and pathology scoring

IHC slides were scanned at × 20 using the Aperio AT2 scanner (Leica). HALO image analysis software (Indica Labs) was used to quantify percentage of tumour nuclei with strong (3+), moderate (2+), weak (1+) or negative pRAD50 staining using a cytonuclear algorithm and tumour tissue classifier. Results were used to calculate a H-Score as follows: [(%1 + cells) + (%2 + cells * 2) + (%3 + cells * 3)]. Classifiers and image analysis algorithms were developed for each cell or xenograft model using a representative training set of images. Human tumour tissue microarrays (TMA)s for triple-negative breast cancer (TNBC), glioblastoma multiforme (GBM), colorectal cancer (CRC) and gastric cancer obtained from Tristar Technology Group, were IHC stained for pRAD50 then evaluated by a pathologist for percentage of positive pRAD50 expressing tumour cells.

### Statistical Analysis

One-way ANOVA with Tukey’s multiple comparisons test was used to determine statistical differences between treatment groups. A value of *p* < 0.05 was considered statistically significant. For data containing only two treatment groups, a student’s unpaired *t*-test was used instead (GraphPad Prism).

## Results

### Multiplexed immuno-MRM mass spectrometry reveals dynamic biomarkers of ATM modulation by AZD0156 following DNA damage

**Fig. 1:**
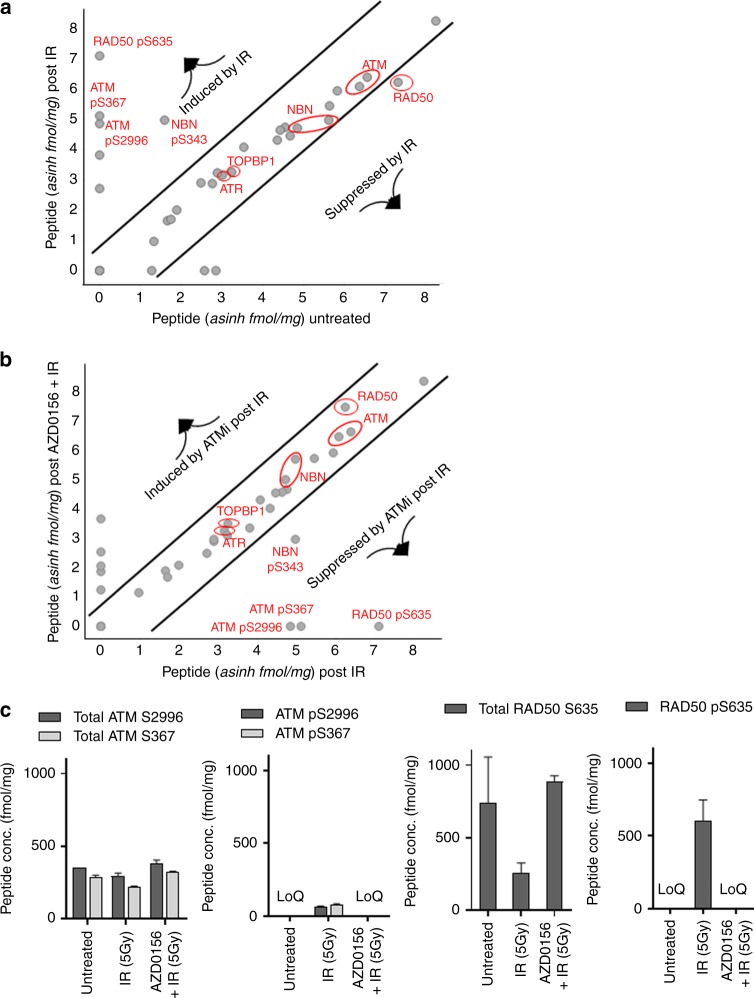
Multiplexed MRM-MS analysis of a panel of DDR related proteins, identifies pRAD50(Ser635) as an ATM-modulated biomarker with high dynamic range. Human PBMCs were either untreated or stimulated ex vivo with 5 Gy of ionizing radiation (IR) in the presence/ absence of AZD0156. **a** Comparison of peptide levels in baseline untreated PBMCs to IR treated PBMCs. **b** Comparison of peptide levels in IR treated PBMCs compared to IR and AZD0156 treated PBMCs. **c** Average concentrations of the total peptide and phosphopeptide analytes in fmol/mg for ATM (S2996 and S367) and RAD50 (S635) given for each treatment group. Peptide levels that were below the limit of quantification (LoQ) are indicated on the graph. See Supplementary Table 2 for LoQ values for each peptide

We applied a 45-plex immuno-MRM-MS assay targeting the DDR signalling network to identify the optimal PD markers of ATM inhibition with AZD0156 in the presence of ionizing radiation (IR). The assay was performed in ex vivo irradiated peripheral blood mononuclear cells (PBMCs), and showed a robust induction of ATM phosphorylation as well as phosphorylation of the MRN components RAD50 and NBS1 in response to irradiation (Fig. 1a), which were inhibited by AZD0156 post irradiation (Fig. 1b). This finding agreed with previous work.^3,29^

The best dynamic range was seen for pRAD50(Ser635), where increased levels of phosphorylated RAD50 were detected following IR that was strongly suppressed by AZD0156 (Fig. 1b, c). RAD50 is known to be phosphorylated by activated ATM at Serine 635, and is essential for SMC1-mediated DNA repair of DSBs.^30^ Assuming a stoichiometric ratio of the total and phosphorylated recovered peptide concentrations, total RAD50 levels were expressed at a consistently high level in the PBMCs, and crucially at more than twice the baseline level of total ATM (Fig. 1c). The dynamic range of formation/suppression of pRAD50 was therefore larger than that for pATM, although both demonstrated complete suppression by AZD0156 (Fig. 1c). The list of quantitative data for the full assay panel are shown in Supplementary Table 1. We note that in this study ATM activation was assessed via phosphorylation of residues Ser367-ATM and Ser2996-ATM.^31,32^ The Ser1981-ATM residue is also commonly assessed for this purpose; however, we were unable to generate a suitable monoclonal antibody for immuno-MRM-MS analysis of the tryptic fragment containing this residue.

### pRAD50 IHC assay development

Clinical tumour tissue is frequently archived in an FFPE format, well-suited to the assessment of relevant biomarker proteins using an IHC assay. IHC staining also has the advantage of preserving spatial relationships between tumour cells and the surrounding tumour microenvironment so biomarker changes can be ascribed to specific cell types. Guided by our findings from immuno-MRM-MS that pRAD50 may have better dynamic range than pATM when interrogating ATM pathway activation, and supporting published work that RAD50 is phosphorylated directly by ATM at Ser635 following DNA damage,^30^ we developed an IHC assay to assess modulation of ATM signalling in tumour tissue using the CST #14223 pRAD50(Ser635) antibody.^33^ Here we report the full development, validation and broader applicability of the pRAD50 IHC assay.

The automated IHC assay successfully showed induction of RAD50 phosphorylation by irradiation in an A549 non-small cell lung xenograft model (Supplementary Figure 2A). Phosphorylation of RAD50 was 4-fold higher in the irradiated xenograft group compared to the vehicle controls (H-score 100 vs. 25 respectively; *P* < 0.001; Supplementary Figure 2B). H-scores were quantified for all xenograft tissues using HALO™ image analysis (Indica Labs), which used a tissue classifier to identify the tumour region and a cytonuclear algorithm to quantify the percentage of 1+, 2+ and 3+ positive tumour cells (Supplementary Figure 2C).

Validation of the pRAD50 IHC assay was carried out as previously described.^34^ Loss of pRAD50 staining after phosphatase treatment of the slides supported the phospho-specific nature of the antibody in xenograft and human tissues (Supplementary Figure 3A). pRAD50 staining was also lost in an irradiated RAD50 stable knock-down cell line further validating the specificity of the assay to pRAD50 (Supplementary Figure 3B). We also confirmed there was no non-specific staining by the detection system by replacing the primary antibody with a rabbit IgG isotype negative control and staining with no primary antibody (Supplementary Figure 3C).

### Moderate baseline pRAD50 levels across different cancer indications

**Fig. 2:**
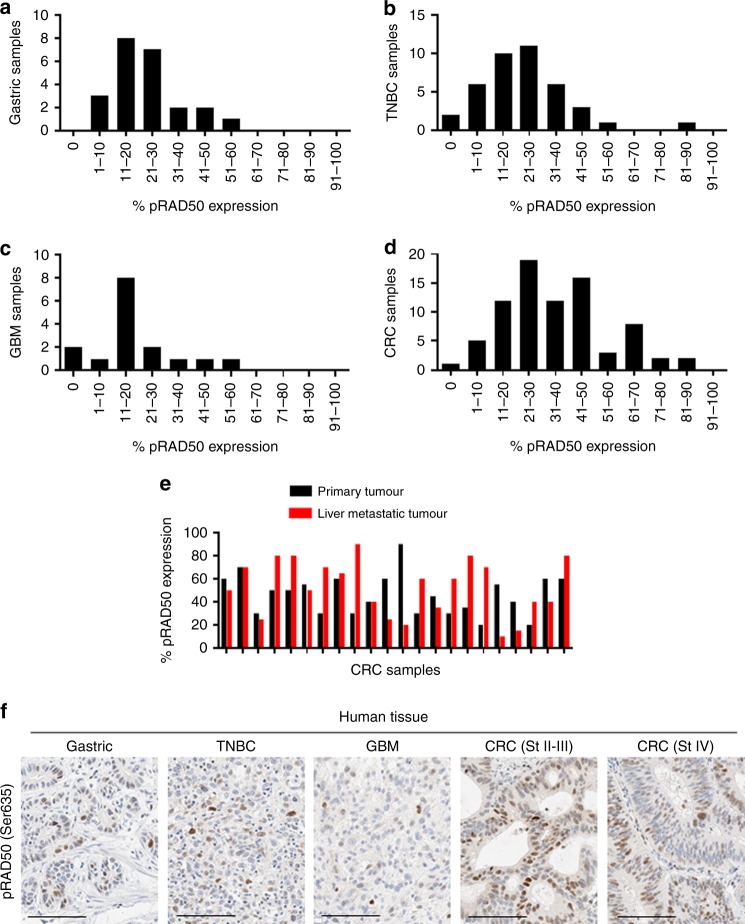
Moderate baseline levels of pRAD50(S635) across different cancer indications. Prevalence of pRAD50(Ser635) protein levels in **a** Gastric cancer; **b** triple negative breast cancer (TNBC); **c** glioblastoma-multiforme (GBM); **d** Colorectal cancer tumours (CRC). Data is given as the number of samples that fall within an expression bin of pRAD50(Ser635) positivity. **e** Percentage of pRAD50(Ser635) expression in matched primary CRC and liver metastatic tumours. **f** Example images of pRAD50(Ser635) IHC staining in each cancer indication that was pathology scored to generate the above data. Scale bars: 100 µm

We considered the possibility that in response to different drug treatment, pRAD50 may be up or downregulated. Therefore, we used the validated IHC assay to assess basal levels of pRAD50 across multiple tumour types, to understand if pRAD50 could be an informative pharmacodynamic biomarker in the clinic. TMAs of gastric cancer, triple-negative-breast cancer (TNBC), glioblastoma-multiforme (GBM) and colorectal cancer (CRC) (Tristar Technology Group) were assessed and pRAD50 was detected in most tissues at a level of 1–50% positive tumour cells (Fig. 2a–f). Although analysis of TMAs cannot well represent heterogeneity of the biomarker across a sizeable tumour, and particularly in our hands the relatively small size of tissue cores in TMAs do not well represent the surrounding tumour microenvironment, it was striking that half of patients showed a more than 25% increase in pRAD50 expression between matched primary and metastatic tumours (Fig. 2e).

Qualitatively, the distribution of pRAD50 across human CRC, TNBC and gastric tumours appear broadly similar with most tumours having less than 50% of cells showing pRAD50 staining although of these tumour types CRC was notable for having a number of tumours where the majority of tumour cells showed pRAD50 staining. In contrast, GBM tumours tended to have lower overall percent cells showing pRAD50 staining. pRAD50 was not detected in 5% of TNBC, 13% of GBM and 1% of CRC patient samples, with all gastric cancer patients’ samples having at least some tumour cells positive for pRAD50. Lack of pRAD50 in these tumours likely indicates lack of endogenous DNA damage in these tissues or loss of MRN function rather than RAD50 loss of function, which is rarely seen in the clinic.^11,12^ There was also a fraction of tumours that had elevated pRAD50 levels (over 50% positive tumour cells). This high baseline level of pRAD50 was observed in 4% of gastric cancer, 5% of TNBC and 6% of GBM, and 24% of CRC patients, and was independent of both tumour grade and disease stage (Supplementary Figure 4). This suggests pRAD50 tumour levels are not prognostic, albeit numbers of low grade and stage tissues were limited. Pathologist review of γH2AX levels in the CRC TMA revealed very low-level gH2AX staining (about 10x less than pRAD50) with no apparent correlation between expression of pRAD50 and γH2AX. Further investigation is needed to determine if the high levels of pRAD50 without γH2AX in these patient tumour samples result from mechanisms of DNA damage and genomic instability distinct from those involved in γH2AX signalling, for example, regressed replication forks vs. frank DNA double-strand breaks.

### pRAD50 IHC assay quantifies modulation of ATM signalling by the ATM inhibitor in combination with irinotecan or olaparib

In an ATM knocked-out FaDu cell line, shown to have no ATM protein expression (Supplementary Figure 5A), irradiation did not upregulate pRAD50 compared to the untreated control when normalised to GAPDH (Supplementary Figure 5B). This contrasted with wild type (WT) FaDu cells, that express ATM protein (Supplementary Figure 5A), and had a strong induction of RAD50 phosphorylation 1 h after DNA damage by IR. This paralleled the responses of other downstream substrates of ATM (pATM and pCHK2) as shown by western blot, with upregulation by IR only observed in the FaDu WT cells, not the ATM KO cells (Supplementary Figure 5C). These data support the use of pRAD50 as an ATM-modulated biomarker, although low baseline levels of pRAD50 in the ATM KO FaDu model suggest there is also an ATM-independent mechanism of phosphorylating RAD50 in the absence of ATM which we will show is ATR.

**Fig. 3:**
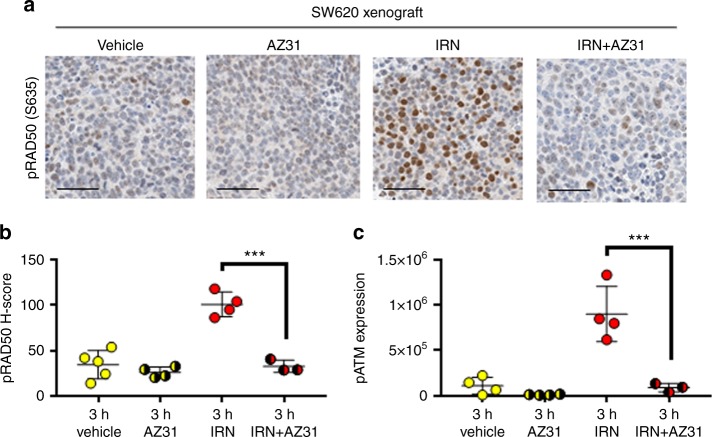
pRAD50(Ser635) quantifies ATM pathway induction by irinotecan and ATM inhibition by AZ31 in an SW620 colorectal xenograft model. **a** Representative images of pRAD50(S635) staining in SW620 xenograft tissue, treated with irinotecan and fixed after 3 h (50 mg/kg intraperitoneal) or AZ31 (100 mg/kg oral) in combination or with a vehicle control. **b** HALO image analysis of the IHC stained samples was used to generate a pRAD50 H-score per sample. **c** Western blot analysis was used to semi-quantify the level of pATM(S1981) protein in each xenograft sample. Mean ± SD given. Each data point indicates an individual mouse. *** indicates *p* ≤ 0.001 by One-way ANOVA, followed by Tukey’s multiple comparison correction. Scale bars: 50 µm

To determine the responsiveness of pRAD50 as a PD biomarker in an in vivo setting, we examined an SW620 CRC xenograft model treated with irinotecan and a probe compound to AZD0156, AZ31.^35^ AZ31 and AZD0156 are both potent and specific inhibitors of ATM, however, AZD0156 was selected as the clinical lead compound subsequent to these experiments when physiologically based pharmacokinetic (PBPK) modelling predicted a longer human plasma half-life for AZD0156 than for AZ31. In this SW620 model irinotecan induced the ATM DNA repair pathway, evidenced by a strong induction of pRAD50 expression post irinotecan (three-fold increase compared to vehicle group; *P* < 0.001; Fig. 3a, b), as well as pATM(Ser1981) induction, quantified by western blot (eight-fold increase compared to vehicle group; *P* < 0.001; Fig. 3c). Addition of AZ31 suppressed the irinotecan-induced phosphorylation of RAD50 and ATM back to the baseline level (68% inhibition of pRAD50; *P* < 0.001 and 90% inhibition of pATM; P < 0.001), demonstrating AZ31 capability to suppress ATM signalling as expected. This data agrees with other work on CRC PDX models, where pRAD50 and γH2AX levels were suppressed by AZ31 post-irinotecan, in a model sensitive to the drug combination.^33^

**Fig. 4:**
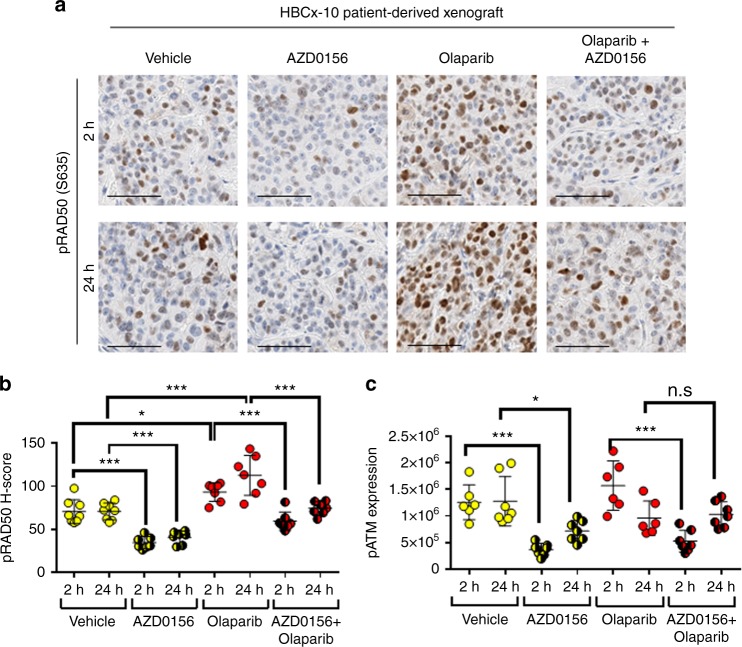
pRAD50 modulation in a BRCA2 TNBC patient-derived xenograft (PDX) model by AZD0156 gives evidence of mechanism of action for the ATM inhibitor. **a** HBCx-10 PDX model was treated with for 3 days with olaparib (50 mg/kg oral qd) or AZD0156 (5 mg/kg oral qd) alone or in combination, and samples were taken at 2 or 24 h post the final olaparib dose. Representative images of pRAD50(S635) IHC staining at each time point are shown. **b** pRAD50(S635) H-score was generated from the IHC stained slides using HALO image analysis. **c** The pATM(S1981) protein level was semi-quantified following western blot analysis of the treated PDX samples. Mean ± SD given. Each data point indicates an individual mouse. * indicates *p* ≤ 0.05, ** indicates *p* ≤ 0.01, n.s. indicates ‘not significant’ by One-way ANOVA, followed by Tukey’s multiple comparison correction. Scale bars: 50 µm

AZD0156, the potent ATM inhibitor currently in phase I clinical trials with the PARP inhibitor olaparib (Lynparza) [NCT02588105], also modulated ATM signalling in a patient-derived BRCA2 mutant TNBC xenograft model (HBCx-10) (Fig. 4). Modulation of the ATM pathway in the HBCx-10 was shown by a 51 and 42% downregulation of its substrate pRAD50, at 2 and 24 h post AZD0156 dosing, respectively (*P* < 0.001; Fig. 4a, b). Quantitative analysis of the H-score for pRAD50 demonstrated a small, but significant, induction of pRAD50 by olaparib, supporting the ability of olaparib to induce ATM repaired DNA damage, which has also been shown in vitro.^36^ Both pATM and pRAD50 levels were reduced by AZD0156 post-olaparib treatment compared to the olaparib monotherapy group (36 and 34% pRAD50 reduction at 2 and 24 h respectively; *P* < 0.001, and 67% pATM reduction at 2 h; *P* < 0.001), supporting the expected mechanism of action of AZD0156.

### ATR inhibition modulates pRAD50 differentially depending on the functionality of ATM

**Fig. 5:**
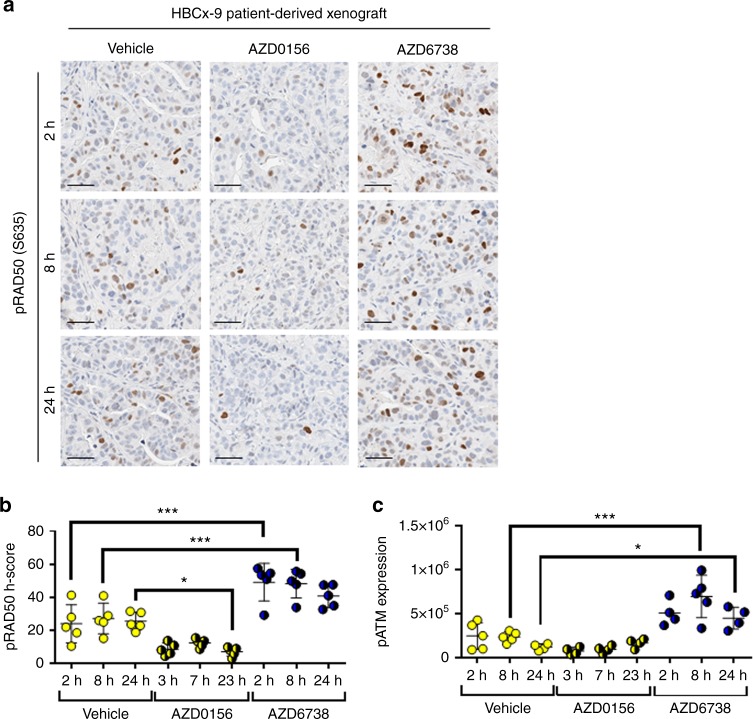
Inhibition of the ATR DNA repair pathway, upregulates ATM kinase activity shown by pRAD50(Ser635) and pATM(Ser1981) induction in a BRCA and ATM WT TNBC patient-derived xenograft (PDX) model. **a** Expression of pRAD50(Ser635) by IHC in a HBCx-9 PDX model post 3 days dosing with AZD0156 (5 mg/kg oral qd) or 5 days dosing with AZD6738 (25 mg/kg qd oral) or the vehicle control. Representative images shown for 3, 7 and 23 h post AZD0156 dose and 2, 8 and 24 h post the AZD6738 or vehicle doses. **b** HALO image analysis was used to generate a pRAD50 H-score per sample. **c** Western blot analysis used to semi-quantify pATM(Ser1981) protein expression in each sample. Mean ± SD given. Each data point indicates an individual mouse. * indicates *p* ≤ 0.05, **p ≤ 0.01, ***p ≤ 0.001 by One-way ANOVA, with Tukey’s multiple comparison correction. Scale bars: 50 µm

In the TNBC PDX model HBCx-9 that is WT for both BRCA1/2 and ATM, AZD0156 monotherapy suppressed pRAD50 expression compared to baseline levels by 72% at 23 h (*P* < 0.05; Fig. 5a, b). There was also a trend towards suppression of pATM by AZD0156 in this model, but this did not reach statistical significance (Fig. 5c). In contrast, treatment with the ATR inhibitor, AZD6738, significantly induced phosphorylation of both RAD50 and ATM (Fig. 5a–c), consistent with findings in the ATM functional NCI-H460 cell line.^16^ In NCI-H460 cells AZD6738 upregulated pATM, stabilised p53, and induced p21 signalling, resulting in the accumulation of cells in the G1 phase and loss of cells from S-phase, highlighting the ability of the ATM repair pathway checkpoints to compensate for loss of the ATR checkpoint control.^16^

**Fig. 6:**
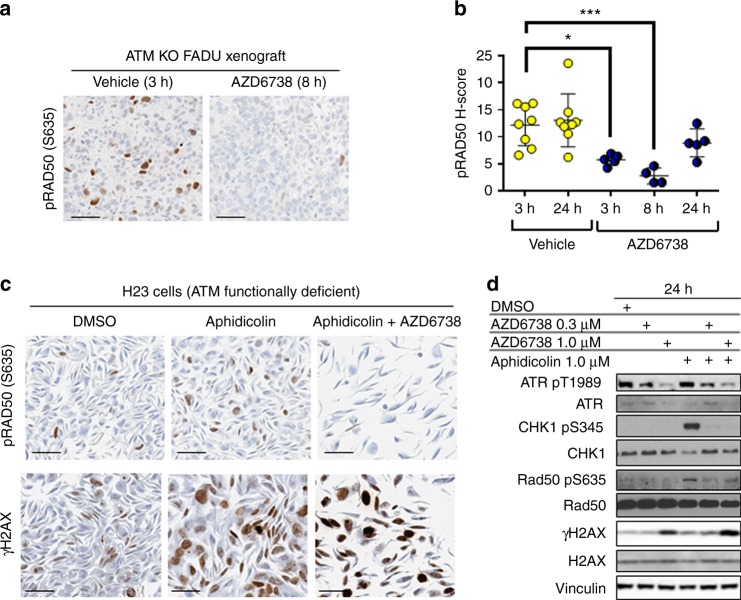
In models with ATM loss of functionality, ATR phosphorylates RAD50; pRAD50 levels are supressed by the ATR inhibitor AZD6738. **a** Expression of pRAD50(Ser635) by IHC in an ATM KO FaDu xenograft model treated with AZD6738 (50 mg/kg oral) or the vehicle control. Representative images are shown for 8 h post AZD6738 dose and 3 h post the vehicle. **b** HALO image analysis was used to generate a pRAD50 H-score per sample. Mean ± SD given. Each data point indicates an individual mouse. * indicates *p* ≤ 0.05, ***p ≤ 0.001 by One-way ANOVA, followed by Tukey’s multiple comparison correction. **c** Expression by IHC of pRAD50(Ser635) and γH2AX in NCI-H23 cells, ATM functionally deficient, 24 h post-treatment with a DMSO control or 1 µM aphidicolin alone or in combination with 1 µM AZD6738. **d** Immunoblot of pATR(Thr1989), pCHK1(Ser345), pRAD50(Ser635) and γH2AX in NCI-H23 cells, 24 h after treatment with DMSO, aphidicolin (1 µM) and AZD6738 (0.3 or 1 µM) monotherapy or in combination. Expression of total proteins also shown for each biomarker normalised to a Vinculin loading control. Scale bars: 50 µm

However, in models where ATM is lost or not functional we observe the opposite effect, as demonstrated by the 32–79% reduction in phosphorylation of RAD50 in the ATM KO FaDu head-and-neck cancer xenograft model treated with AZD6738 for 24 h (Fig. 6a, b). We note that the reduced effect of AZD6738 at the 24 h post-treatment time point is likely attributable at least in part to the considerably faster clearance of AZD6738 in mice vs. humans. Abrogation of pRAD50 signalling by AZD6738 was also seen in ATM functionally deficient non-small-cell lung cancer NCI-H23 cells (Fig. 6c). The NCI-H23 cells have a missense mutation in ATM (Q1919P) that has been shown to prevent ATM signalling.^16^ Treatment of NCI-H23 cells with a combination of aphidicolin and AZD6738, induced high levels of DNA damage, evidenced by the elevation in pan-nuclear γH2AX (Fig. 6c). In contrast, pRAD50 levels were reduced by AZD6738 in the presence of aphidicolin, compared to aphidicolin treatment alone (Fig. 6c). Consistent with the IHC data, western blot quantification of NCI-H23 cells treated with the combination of AZD6738 (1 uM) and aphidicolin showed a 56% reduction in pRAD50 and 1.5-fold increase in γH2AX, compared to aphidicolin treated cells. Inhibition of ATR was demonstrated by a 33% decrease in pATR and ablation of phosphorylation on its downstream substrate CHK1 (Fig. 6d).

## Discussion

Clinically amenable assays of ATM pathway inhibition will help inform ongoing clinical and translatable preclinical studies involving ATM inhibitors such as AZD0156, and more broadly in studies of DNA damage and repair. Although inhibition of ATM phosphorylation represents the most proximal PD marker for ATM inhibition, our initial attempts to develop an IHC assay were limited by a lack of sensitive and specific antibodies to phosphorylated ATM (Supplementary Table 2). Notwithstanding the literature reports of pATM IHC with one of these antibodies,^37,38^ in our hands none passed the full suite of validation assays we require for clinical deployment. We therefore sought to identify additional markers of ATM inhibition that could be robustly modulated by AZD0156 and measured by IHC.

We used immuno-MRM-MS to guide the selection of pRAD50 as an alternative biomarker for interrogating the ATM pathway signalling and subsequently developed a pRAD50 IHC assay for clinical use. Immuno-MRM-MS has demonstrated the ability to generate rapid and precise quantification of signalling networks through highly specific measurement of total and phospho-peptides,^24,26,39^ although it must be noted that immuno-MRM-MS is necessarily limited by both the availability of peptide-enriching antibodies and by the amenability of the various protein tryptic fragments to MS analysis. Interestingly the immuno-MRM-MS methodology is sufficiently sensitive to quantify the low concentration of ATR in PBMCs, with baseline ATR peptide concentrations approximately 30-fold lower than those of ATM peptides, consistent with published reports of very low/undetectable baseline ATR levels in PBMCs.^40^ Since PBMCs are easily accessible in clinical trials, there is also potential to implement the immuno-MRM-MS in the clinical setting for analysis of surrogate PD biomarkers.

Using our validated pRAD50 IHC assay we assessed the suppression of DNA-damage-induced ATM signalling by AZ31 and AZD0156 ATM inhibitors, in 2 preclinical xenograft models. These results were in agreement with other in vitro and in vivo findings from our group,^33,36^ and were consistent with the expected mechanism of action. We also showed in Gastric cancer, TNBC, CRC, GBM, a variability in baseline pRAD50 levels between patients. On average pRAD50 expression was modest, with most patients expressing detectable levels of pRAD50. This means there is scope to measure both up and downregulation of RAD50 activity following a variety of experimental stimuli. Further, we observed significant variation of pRAD50 levels between matched primary and liver metastatic CRC tumours. Differing expression of biomarkers have been previously reported between primary and metastatic sites for markers such as EGFR and Ki67,^41,42^ but such data has not been previously reported for RAD50 activity. These findings highlight the importance of taking paired biopsies from the same tumour site to reduce the impact of tumour site heterogeneity on PD assessment. In addition, clinical trial paired biopsies should ideally be processed in a well-defined manner and fixed immediately to reduce preanalytical variables, such as formalin fixation delay which is known to be detrimental to preservation and assessment of phospho-markers using FFPE IHC. A potential limitation of this current work is the unknown degree of sample preparation variability associated with production of commercially sourced TMAs.

The prognostic value of pRAD50 has yet to be investigated, but studies have used γH2AX to associate high basal levels of DNA damage to worse prognosis in a number of cancer indications, including TNBC, non-small cell lung cancer and CRC. Elevated γH2AX was also linked to more malignant cancer behaviour.^43^ Despite lack of prognostic data on pRAD50, increased RAD50 copy number in breast cancer patients was associated with reduced overall survival (OS), and correlated to expression of total RAD50 at the RNA level.^44^ Similar findings have been found for the other members of the MRN complex, with increased expression of MRE11 and NBS1 associated with shorter survival in CRC,^45^ high expression of MRE11 combined with ATM associated with lower OS and disease-free survival in rectal cancer,^35^ and high levels of NBS1 correlating with lower recurrence-free survival in EOC.^46^ Not all studies agree, however, with some showing no prognostic value of MRE11 in anal cancers treated with chemo-radiotherapy,^47^ and favourable prognosis of high NBS1 in pancreatic cancer,^48^ highlighting differences in prognostic biomarkers between cancer types and different therapy regimens. It would be interesting to investigate further whether pRAD50 could be used as a prognostic marker as well as a PD biomarker and we note the additional possibility of interrogating pRAD50 on circulating tumour cells (CTCs)^49^ as a potential clinically amenable surrogate tumour tissue assessment method.

Whilst ATM and ATR are activated by distinct types of DNA lesions, DNA-processing and inter-conversion of DNA substrates can lead to complex interactions between the ATM and ATR signalling pathways,^50^ as well as significant overlap between downstream kinase targets to maintain genome stability.^29^ This cross-talk between ATM and ATR substrates is likely to result from changes to DNA end processing, which can convert lesions to be recognisable by either kinase.^50^ In the case of RAD50 recent findings show phosphorylation at Serine 635 is not only essential for ATM signalling, but is also required for ATR activation in response to DNA replication stress, enabling replication fork restart and ATR signalling via CHK1.^51^ Our results are consistent with the hypothesis that in the absence of ATM, RAD50 can be directly phosphorylated by ATR,^51,52^ presumably as a compensatory mechanism. Furthermore, the difference in pharmacodynamics of pRAD50 following treatment with AZD6738 in ATM functional and dysfunctional models, suggests the pRAD50 IHC assay may have the potential to ascertain ATM functionality in preclinical models and patients treated with AZD6738. Studies to test this are currently ongoing. Together these data support the clinical utilisation of pRAD50 as a biomarker of both AZD0156 and AZD6738 pharmacology in either monotherapy or combination settings.

To our knowledge this is the first time multiple-reaction-monitoring mass-spectrometry has been used to identify a PD biomarker for clinical deployment. We demonstrate the utility of a multiplexed immuno-MRM-MS assay for identifying PD markers of kinase inhibition and describe how this led to the development and validation of a novel clinically applicable IHC biomarker assay against pRAD50. We show the ATM inhibitors (AZ31 and AZD0156) abrogate phosphorylation of RAD50, consistent with the expected mechanism of action for these compounds. Whereas the ATR inhibitor AZD6738 was also able to modulate phosphorylation of RAD50, but in a context-dependent manner, highlighting the potential to use pRAD50 modulation to determine ATM functionality. Furthermore, we found sufficient baseline levels of pRAD50 in clinical tumour samples to enable the pRAD50 IHC assay to be deployed clinically and facilitate recommended phase 2 dose selection.^20^ Future consideration will also be given to deployment of an immuno-MRM assay for clinical use, where published fit-for-purpose validation guidelines^53^ and best practices for handling critical assay reagents^24^ have laid the foundation for a regulatory path to be established.

## Electronic supplementary material

Supplemental Figure 1

Supplemental Figure 2

Supplemental Figure 3

Supplemental Figure 4

Supplemental Figure 5

Supplemental Table 1

Supplemental Table 2
