# Supplementary figures and images for: pRAD50: a novel and clinically applicable pharmacodynamic biomarker of both ATM and ATR inhibition identified using mass spectrometry and immunohistochemistry
Source: Br J Cancer. 2018 Nov 2;119(10):1233–43. doi: 10.1038/s41416-018-0286-4 (PMC6251026; doi:10.1038/s41416-018-0286-4)

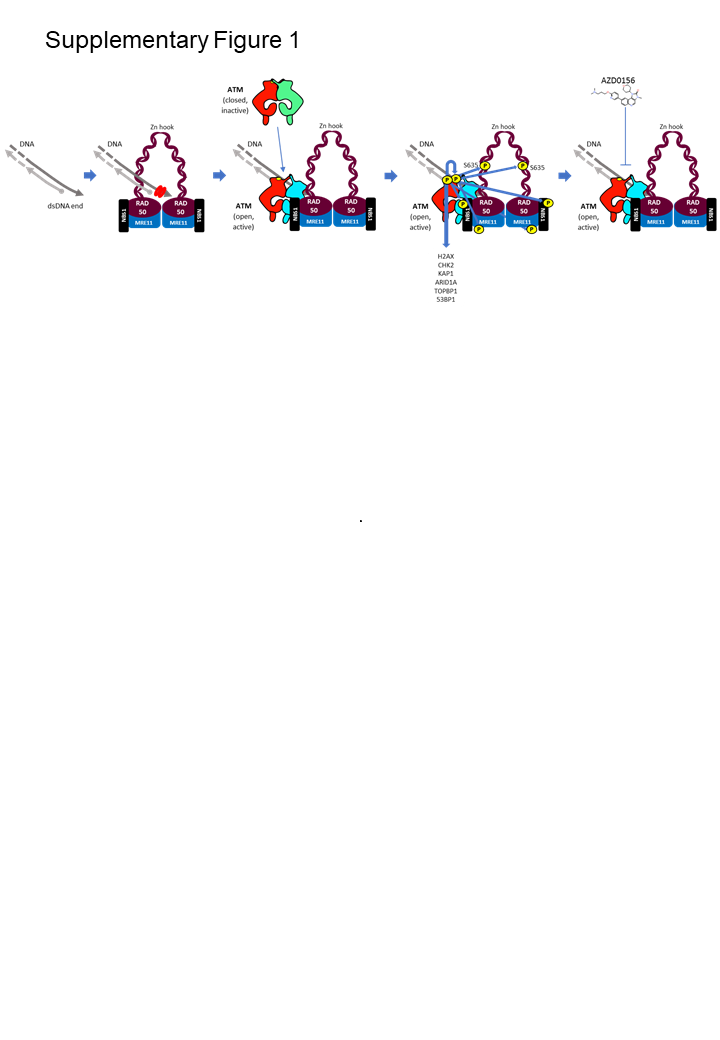

Supplement: Supplementary file 1 — Supplemental Figure 1 [file 41416_2018_286_MOESM1_ESM.tif]

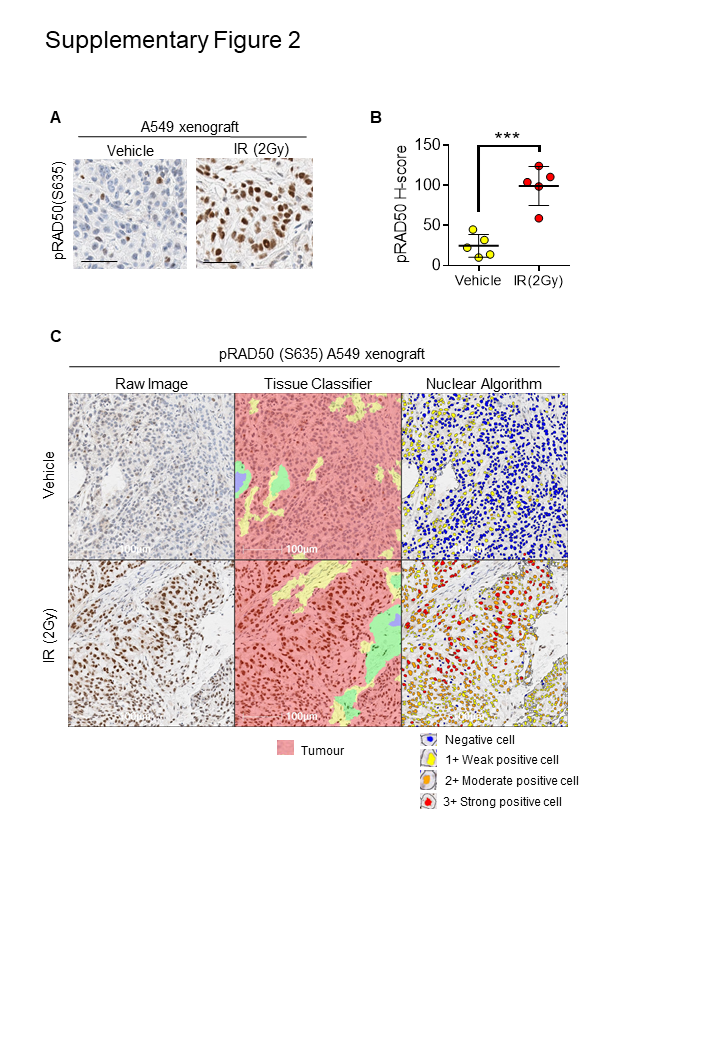

Supplement: Supplementary file 2 — Supplemental Figure 2 [file 41416_2018_286_MOESM2_ESM.tif]

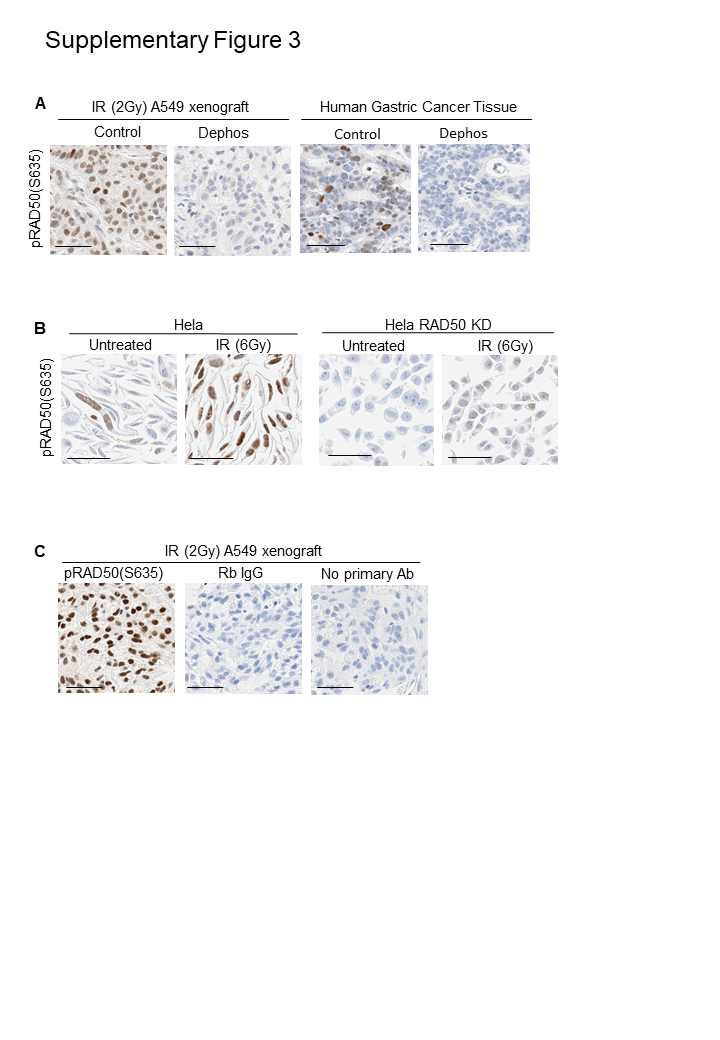

Supplement: Supplementary file 3 — Supplemental Figure 3 [file 41416_2018_286_MOESM3_ESM.tif]

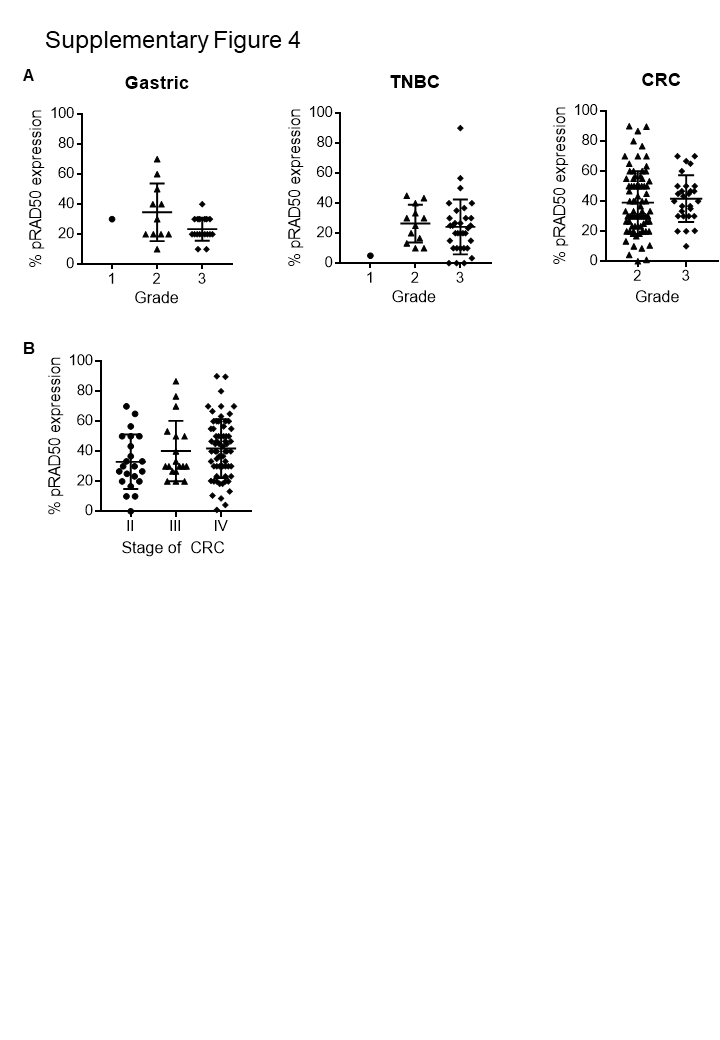

Supplement: Supplementary file 4 — Supplemental Figure 4 [file 41416_2018_286_MOESM4_ESM.tif]

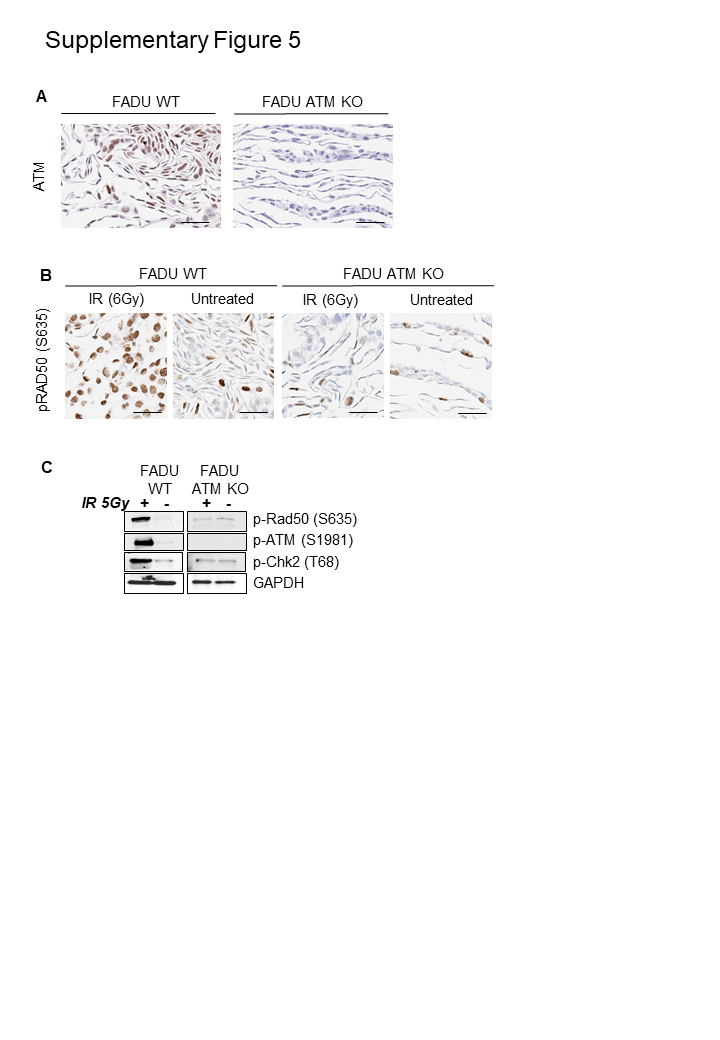

Supplement: Supplementary file 5 — Supplemental Figure 5 [file 41416_2018_286_MOESM5_ESM.tif]

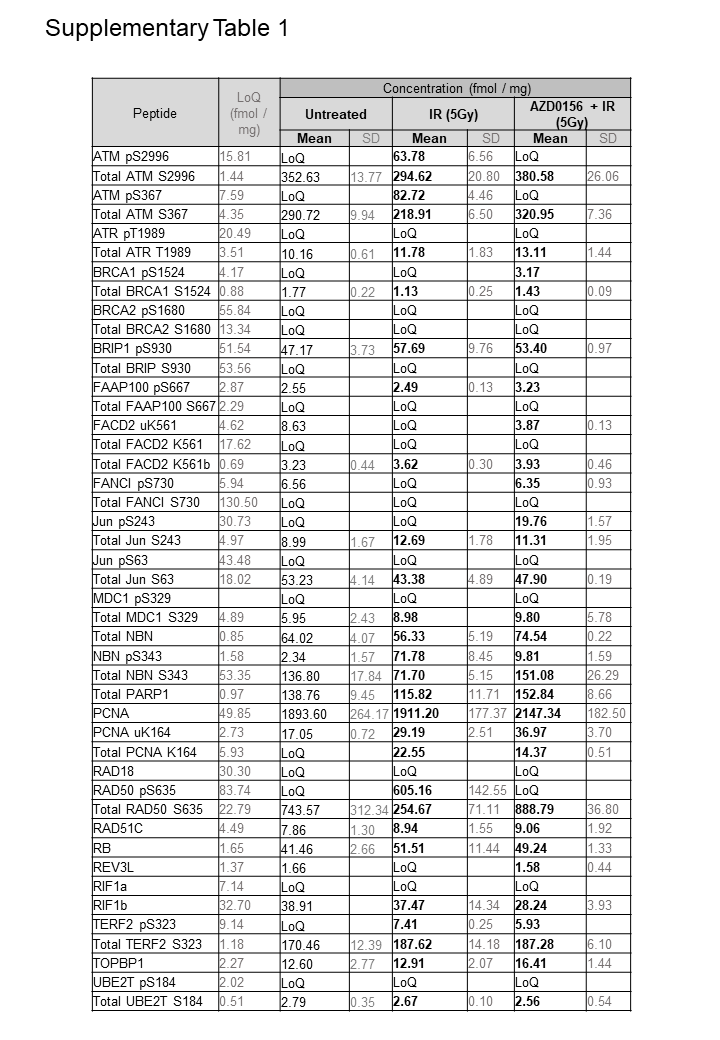

Supplement: Supplementary file 6 — Supplemental Table 1 [file 41416_2018_286_MOESM6_ESM.tif]

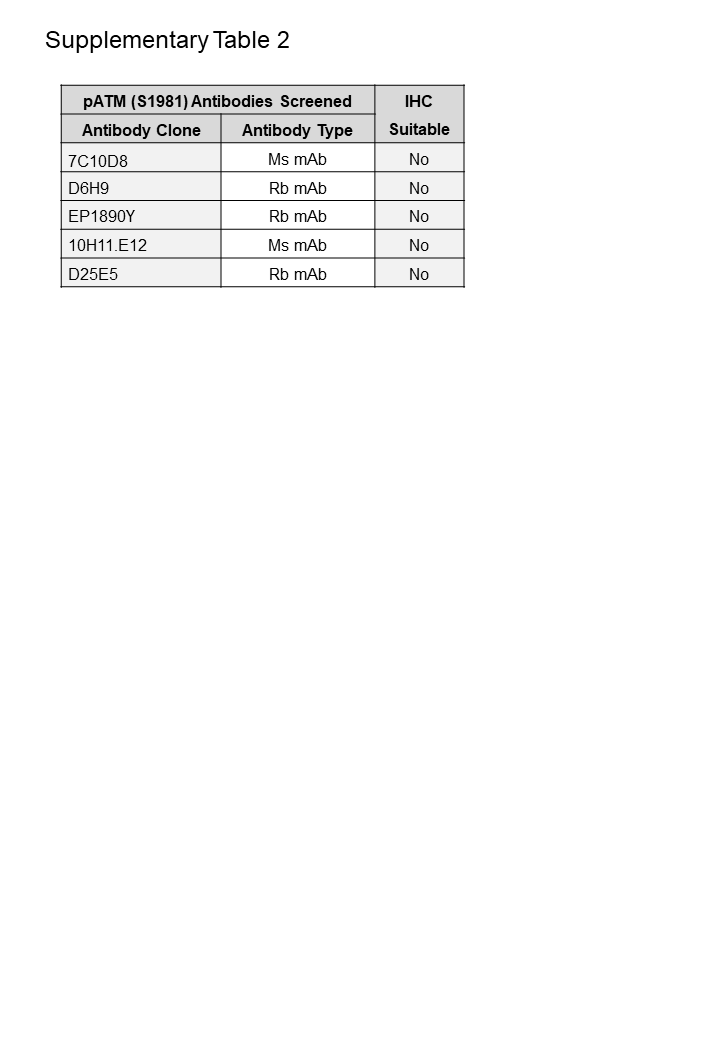

Supplement: Supplementary file 7 — Supplemental Table 2 [file 41416_2018_286_MOESM7_ESM.tif]
